# The Renovation of Good Clinical Practice: A Framework for Key Components of ICH E8

**DOI:** 10.1007/s43441-023-00561-x

**Published:** 2023-12-01

**Authors:** Madeleine Whitehead, Melissa Suprin, Tashan Mistree, Margaret Mary Kearns, Gerard Marini, Christine Goffe, Marion Pillwein, Vimi Abdul-Shukkoor

**Affiliations:** 1 Roche, Hexagon Place, Shire Park, Falcon Way, Welwyn Garden City, AL7 1TW UK; 2 Pfizer, 280 Shennecossett Rd, Groton, CT 06340 USA; 3 GSK, 1250 S Collegeville Rd, Collegeville, PA 19426 USA; 4 MSD, 120 Moorgate, London, EC2M 6UR UK; 5 Sanofi, Corporate Dr, Bridgewater, NJ 08807 USA; 6 UCB, Chem. du Foriest 1, 1420 Braine-l’Alleud, Belgium; 7 BMS, Rte de Perreux 1, 2017 Boudry, Switzerland; 8 Eli Lilly & Company, 33 Imclone Dr, Branchburg, NJ 08876 USA

**Keywords:** Quality by design, QbD, Critical to quality factors, CtQ, E8, Stakeholder engagement, Critical thinking, Open dialogue, Fit for purpose

## Abstract

The International Council for Harmonisation of Technical Requirements for Pharmaceuticals for Human Use’s (ICH) renovation of Good Clinical Practice (GCP) represents a philosophical shift in the conduct of clinical research away from a one-size-fits-all application to promoting a proactive, risk-based approach. The aim of this paper is to enhance the understanding of specific topics detailed in ICH E8 based on direct feedback from TransCelerate member companies who identified Quality by Design (QbD), Critical to Quality (CtQ), Fit for Purpose, and Stakeholder Engagement, as most changed and open to interpretation. The TransCelerate framework seeks to highlight and expand each of these central topics to support utilization and implementation of a strong foundation for quality in clinical development.

## Introduction

**Figure 1:**
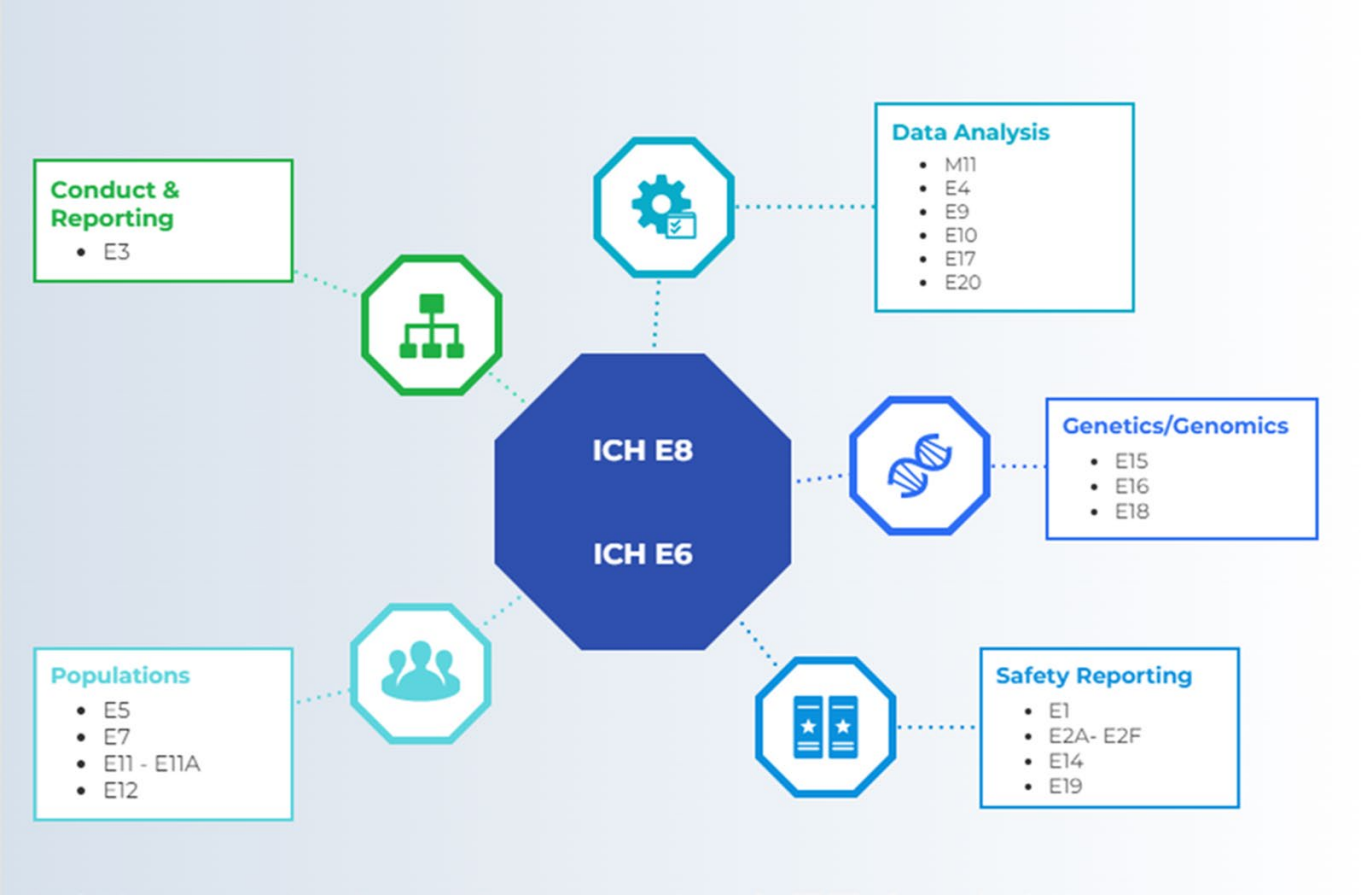
Integration of ICH E8 with other guidelines. See “Appendix 1” for titles of guidelines.

The ICH’s renovation of GCP embraces the innovations in the conduct of clinical research over the last 25 years. ICH E8 is an overarching document; the scope spans from the inception of drug development plans to the reporting of clinical study results to regulatory authorities. Although embedded in the Efficacy segment of ICH Guidelines, ICH E8 sets the foundation for conducting clinical development with quality (Fig. 1).

The aim of this paper is to enhance the understanding of specific topics detailed in ICH E8 based on direct feedback from TransCelerate member companies who prioritized Quality by Design (QbD), Critical to Quality (CtQ), Fit for Purpose, and Stakeholder Engagement as most changed and open to interpretation. The TransCelerate framework seeks to highlight and expand each of these central topics to support a strong foundation for quality in clinical development, while looking to the future.

**Figure 2:**
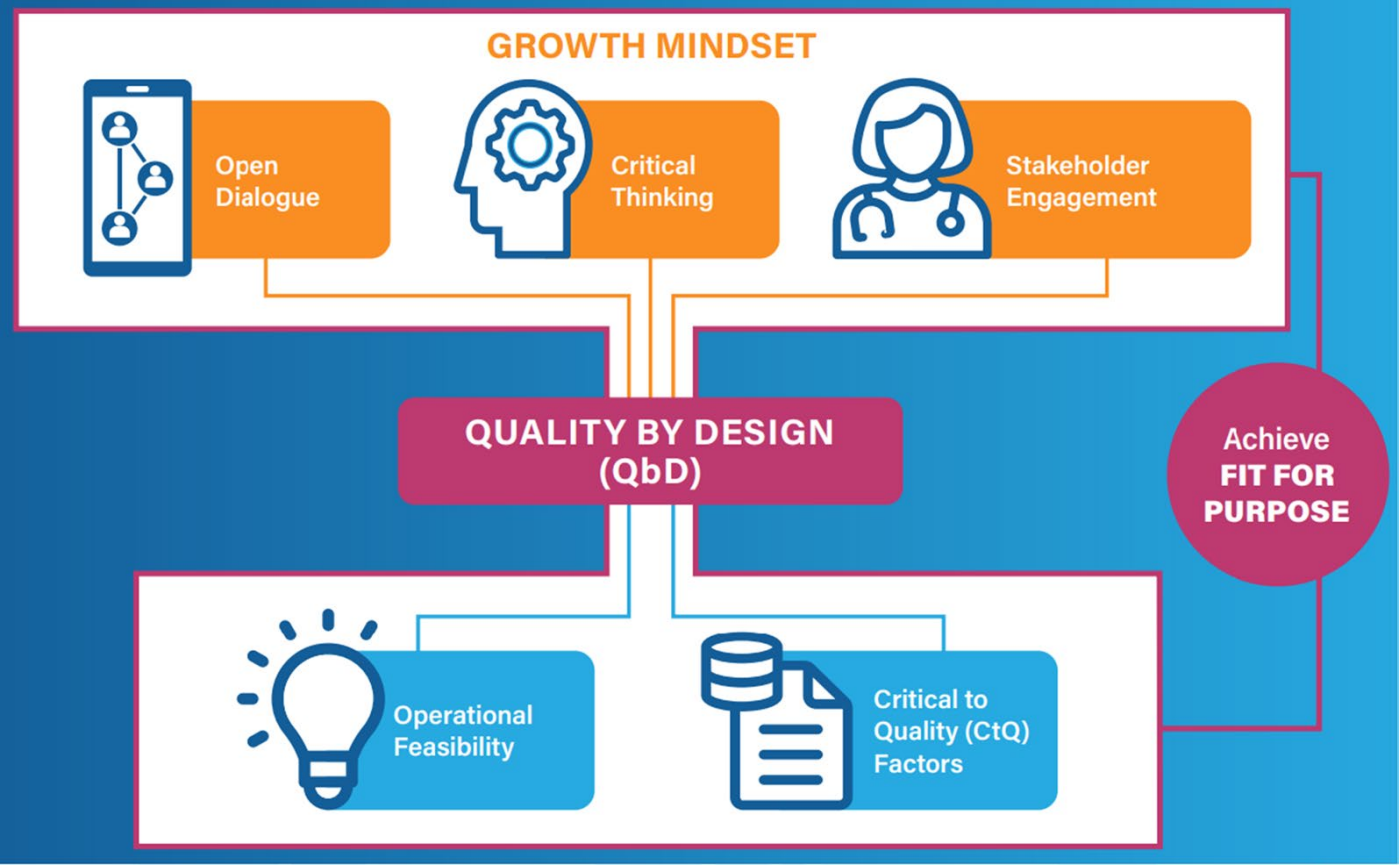
TransCelerate: what you need to know about ICH E8.

This Paper groups linked concepts together under the headings of “Designing Quality into Clinical Studies” and “Culture and Engagement,” mirroring the holistic themes that run through ICH E8 (Fig. 2).

## Designing Quality into Clinical Studies

### Fit for Purpose

ICH E8 now incorporates the concept that quality in clinical studies should be fit for purpose. That is, quality is most effective when applied in a way that aligns with the study intent and design. This customized approach to quality can be associated with all components of clinical development including the design and management of drug development programs and clinical studies. One way to achieve a fit for purpose approach is to apply quality by design principles enabling a proportional allocation of resource based on what is critical to quality.

### Quality by Design

The premise of quality by design is simple: incorporate quality in the design from the beginning and focus on what matters to quality. ICH E8 describes quality by design for clinical development in that same spirit, reducing risks and issues for the benefit of study participants and the reliability of study results.

Defining quality underpins any approach to quality by design. ICH E6 and ICH E8 describe quality in a variety of ways; each connects to the protection of study participants’ rights, safety, and well-being and the reliability and interpretability of study results. These are core to the quality of clinical research activities across many stakeholders. Other factors that could be included in the definition of quality are company-specific or related to specific aspects of quality and compliance. Whatever quality definition is used becomes the basis for other elements of the quality management system, including the application of quality by design concepts.

Quality by design supports quality in all clinical programs including those which rely on real-world data and/or large interventional studies. The first step in applying a quality by design approach is defining factors that are critical to quality: those attributes that matter most to the clinical study outcomes, and for which errors cannot be tolerated without negatively impacting a quality outcome. Using critical to quality factors not only defines what matters most but also enables communication and the application of risk-proportionate controls across the Quality Management System (QMS).

Resources for supporting the implementation of successful quality by design are available in the TransCelerate *Culture and Engagement Resources Pack*.

### Critical to Quality

ICH E6 expressed critical to quality factors as “critical processes and data.” ICH E8 takes a broader definition, widening the category of critical to quality factors to include any that may impact participant protection or the reliability and/or interpretability of trial results. For those who have already implemented ICH E6, critical processes and data are still useful to maintain, but now with additional factors to consider. ICH E8 emphasizes defining critical to quality factors early in clinical development planning and then periodically throughout the related studies. In addition, reassessment of critical to quality factors may be warranted when there is:

- A significant change to the clinical development plan or to a study design, which may be indicated by a revision to the clinical development plan or a protocol amendment;
- A significant or repeat quality issue/event;
- A trend in quality performance;
- An external factor that affects quality or quality controls;
- A long period between study milestones.

A broad group of stakeholders can have input to defining what is critical to quality from their perspectives. This is described further in the “Stakeholder Engagement” portion of this Paper.

Critical to quality factors can be defined using a number of methods, including real-time or synchronous brainstorming sessions with stakeholders and use of critical to quality trees or other tools that support team decision making. Additional information is available in the TransCelerate *Resources for the Application of Critical to Quality Factors*.

**Figure 3:**
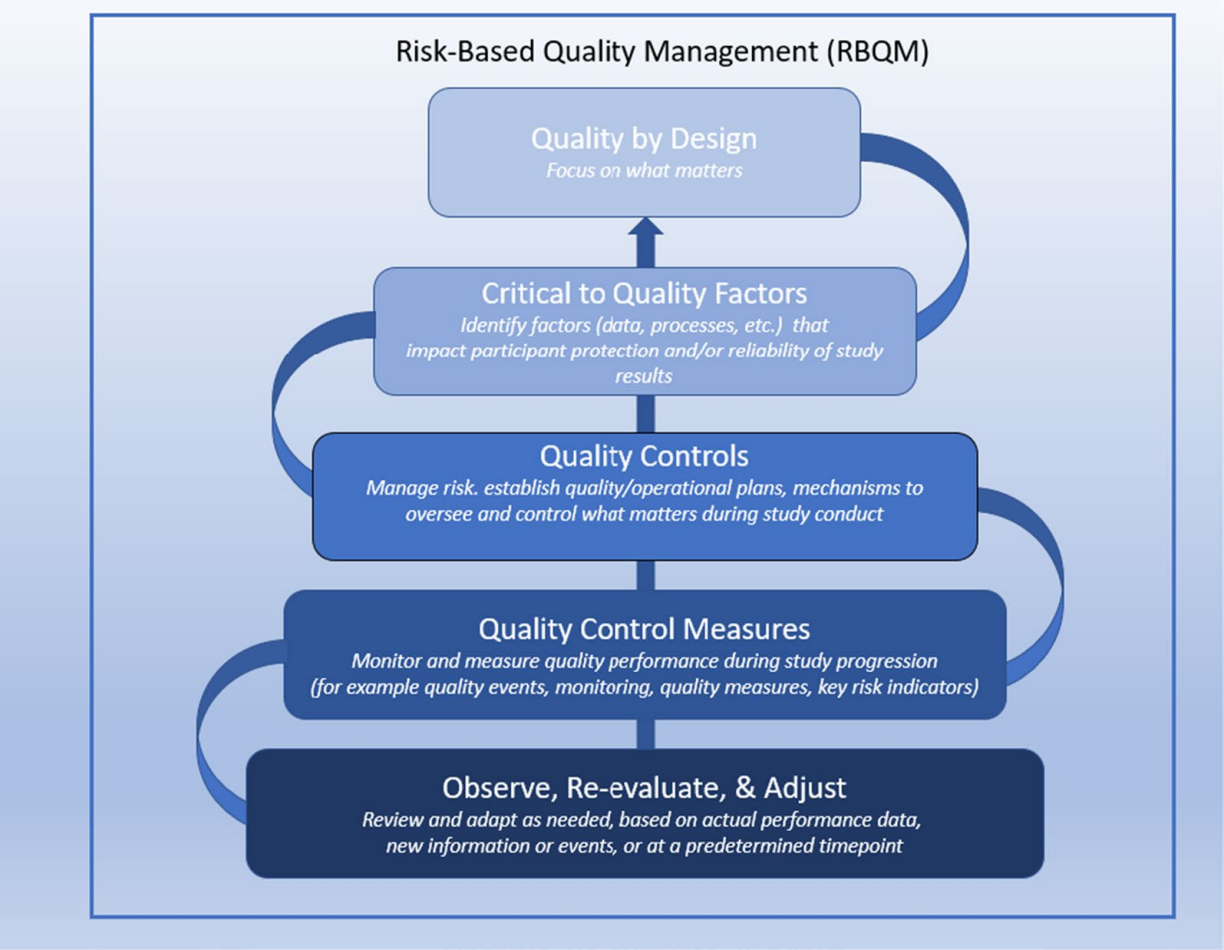
TransCelerate quality by design key components.

ICH E8 section 7 lists possible critical to quality factors that can be used as a guide to inspire brainstorming or simply as a list of factors to consider. The list of critical to quality factors in section 7 is neither exhaustive nor a limit on which critical to quality factors may be used, nor is it a requirement to use all those listed. If all the factors identified on the list were applied to one clinical study, it would be difficult to determine and communicate what was important to quality, thereby defeating the objective of focusing on what matters most. Whichever method is employed to determine critical to quality factors, factors should be limited to those that matter *most* to quality, the implementation of the clinical study, participant protection and safety, the organization, and for the particular circumstance (Fig. 3).

### Operational Feasibility

The foundation of a successful study is a protocol that is both scientifically sound and operationally feasible.^1^

ICH E8 now highlights operational feasibility as a factor that directly affects quality. Operational feasibility promotes efficient and effective application of the protocol with quality and can be achieved by asking questions when identifying critical to quality factors, including:

- What is essential to achieve the study objectives?
- Are there operational limitations which impact what can be achieved and require the revision of critical to quality factors identified during study design?
- Can sites and investigators (e.g., equipment, facilities, training, and qualifications) implement the protocol?
- Are there country or site-specific differences or limitations?
- What is the availability of participants based on the target patient population?
- What are the risks in conducting the study, how can these be eliminated or managed through study design?
- How can burden for sites, investigators and participants be minimized?
- Are there challenges with retention and follow up of study participants?

While operational feasibility is not a new concept and currently many of these questions are being asked, ICH E8 expands on the “who” is being asked. ICH E8 advocates engaging with much broader and more diverse stakeholders like regulators, patients and patient advocacy groups, and other experts, and having those discussions as early as clinical development planning.

#### What Could “Good” Look Like?

Designing quality into clinical studies, as described above, is a prospective activity with the purpose of refining the study or development plan to generate accurate answers to clinical questions and to protect participants from harm. ICH E8 describes how the concepts could be applied to prioritize the elements that matter the most to the quality of the study and to streamline non-essential activities in study conduct and oversight. This means moving away from “inflexible, one-size-fits-all approaches”^2^ dependent on retrospective quality control, audits, and inspections to detect errors.

At a minimum ICH E8 recommends the use of quality by design principles to create an operationally feasible and scientifically sound protocol based on the identification of critical to quality factors for associated risks and the implementation of appropriate controls to provide oversight of those factors. Designing quality into the a clinical study is not completed when the protocol is drafted, it should be a continuous and ongoing pursuit throughout the study lifecycle, building and course correcting the study design and the critical to quality factors based on knowledge and data gained during study conduct.

Where risk-based quality management is fully implemented the concepts of risk management (ICH E6^3^: identification, evaluation, control, review, and reporting) are intertwined with the key elements of ICH E8.

What could good look like for designing quality into clinical studies?

**Table:** 

How and what	Purpose
During clinical planning identify risks associated with critical to quality factors	The clinical plan can be altered to eliminate or reduce the risk and to focus on what matters most For example, streamlining the number and type of studies and the being data collected could increase operational feasibility, and reduce burden for participants
Evaluate risks identified during study design	Critical to quality factors identified during design can be evaluated to determine appropriate risks and controls, including where applicable, the data to be collected, the measures, and the analytical approaches to be applied
Apply concepts of risk-based quality management to create appropriate study conduct strategy	Assess the processes involved in the conduct of the study to evaluate if they are essential to the outcome of the study. For those determined as critical, add measures to control those processes (e.g., edit checks, error-proofing site-based processes)
	Assure that the data collected is of quality and addresses the question; address and limit missing data; Streamline data collection points and queries to remove those which do not support the critical to quality factors (e.g., prioritizing Protocol Deviations, quality events, and audits based on critical to quality factors)
	Create the appropriate feedback loops, allow course corrections to be made when errors arise, and enable resources to be focused and refocused on what matters based on data and knowledge (e.g., monitoring, management, and oversight)
Eligibility Criteria	Create eligibility criteria which reflects the population with the disease or condition in the real world. Apply a proportionate approach to determine broad eligibility criteria to ensure thorough assessment of the safety and effectiveness of the study drug
Communication with Service Providers [i.e., Contract Research Organizations (CROs)]	Include the operational team, including the CRO and other Service Providers, in the study design process and in the identification of CtQs
Oversight Mechanisms	Adopt proactive mechanisms such as metrics, data analysis, and data review focused on the critical to quality factors to demonstrate oversight

ICH E8 acknowledges the increased complexity and changing nature of clinical studies and aims to provide direction for identifying and measuring errors during study conduct in order to understand and focus on what matters most. What quality looks like can and should be different from organization to organization and study to study.

## Culture and Engagement

### Culture of Open Dialogue

ICH E8 emphasizes the important role that open dialogue has in the effective implementation of quality by design principles and practices. Open dialogue builds relationships and trust among stakeholders and facilitates decision-making. It enables robust study design and the identification of critical to quality factors. A fit for purpose outcome cannot be achieved without a culture of open dialogue.

The concept of open dialogue can be applied among many stakeholders and, like all aspects of culture, is reflected in the behaviors of an organization, for example, one that rewards transparency and inclusion. Table 1 provides examples of the behaviors associated with a culture of open dialogue:

**Table 1:** Examples of creating a culture of open dialogue

A culture of open dialogue	A culture without open dialogue
Study participants are included in the conversation about the risks and benefits of their clinical study	The conversation about the risks and benefits of participating in a clinical study is a one-way communication, too long, or too hard to understand
Risks are identified by all participating in study activities without negative consequences and in the spirit of protecting study participants, ensuring data integrity and preventing additional work	Presenting a risk is associated with blame, extra work, and negative perceptions of the individual or group identifying the risk
Stakeholders are engaged for their expertise and input to determine specific critical to quality factors for the study design	Risks could be copied from prior studies without consideration of changes or are treated as required but not useful
Issues are addressed in the interest of process improvement and learning. Raising issues is welcomed and encouraged	Issues are associated with blame, extra work, and negative perceptions of the individual or group identifying the issue
Regulators are engaged as trusted partners; as such, data and information are shared proactively, openly, and honestly	Minimal information is shared with regulators as required to progress an inspection or submission

### Critical Thinking

ICH E8 now encourages critical thinking based on knowledge (e.g., disease area knowledge, treatment knowledge, regulations, local knowledge, and participant knowledge), data, and a deep understanding of the clinical study to take thoughtful, strategic decisions that are consistent with participant protection and the reliability and interpretability of study results.

Critical thinking encourages proactive, collaborative discussions and actions focused on what matters for a clinical study or for the development program. The application of critical thinking can inspire the development of innovative methods or support development of risk-based approaches. ICH E8 recommends creating and utilizing quality measures to assess and reward critical thinking. For example, rewarding robust risk management plans and error-free study execution.

Critical thinking can be used during study design, building quality into the study proactively, identifying critical to quality factors and reducing potential risks and issues. Critical thinking is an ongoing pursuit, undertaken daily to identify critical to quality factors, manage risks, incorporate stakeholder input, and ultimately run a fit for purpose study.

Critical thinking is a multifaceted endeavor that may include:

**Observation**: Identifying opportunities, risks, and solutions
**Analysis**: Gathering and interpreting data and information to question, identify patterns, and make decisions
**Communication**: Sharing and receiving information using varying methods (written, verbal, non-verbal) with multiple and differing stakeholders, on an individual basis, or in a group setting
**Problem solving**: Identifying problems; bringing people, data and information together, assessing the problem creating and implementing a pragmatic solution
**Open mindedness**: Challenging assumptions or judgements to create a strategy or position.

When making decisions throughout the life of a study or development program applying critical thinking increases the likelihood of success.

### Stakeholder Engagement

Engaging internal and external stakeholders supports achievement of operational feasibility, the scientific objectives, and the application of fit for purpose quality controls.

Beyond the cross-discipline inputs from within the sponsor organization, clinical development planning benefits from input from informed stakeholders such as clinical investigators, study coordinators, participants, external service providers, Contract Research Organizations (CROs), Independent Review Boards (IRBs), and Regulators. Each stakeholder enhances quality by design by providing valuable insights.

ICH E8 encourages initiating interactions, discussions and the sharing of knowledge with all stakeholders early in the development of the clinical development plan and/or study. In addition, stakeholder input can be valuable when there are significant changes, including but not limited to the addition of a new arm to the clinical study, a change to the dosing regimen, substantial protocol amendments, and a change of sponsor due to acquisition.

For studies with novel features, engagement with key stakeholders and regulators is critical at an early stage. This may help define study populations and procedures.

We have seen the benefits of early engagement with the MHRA (and regulators in general) and would encourage Sponsors to come to us as early as possible for regulatory advice. This is an age of change, adopting new technologies, decentralised trials, novel trial designs and encouraging diversity in clinical trial designs.^4^

In summary, stakeholder engagement supports effective clinical development and study planning, management, and quality. Further information and guidance are located in the TransCelerate *Stakeholder Engagement Resources.*

#### What Could “Good” Look Like?

Collaborating with patients and patient advocacy groups early in the development of the study design has tangible benefits for all parties. Sponsors better understand what really matters to patients and patients’ caregivers, manage risks before initiating study conduct and establish trust. Patients establish a connection to care and make a valuable contribution to the knowledge and advancement of treatment options.

What could good look like for patient and patient group engagement?

**Table:** 

How and what	Purpose
Informing research priorities For example: Insights into what matters most to patients Insights into living with a disease/condition Insights into patient treatment options	Engaging with patients and caregivers early during the study concept stage to identify and incorporate what matters most enables the development of more efficient and effective studies
Review and provide input to study protocols For example: Number and difficulty of assessment visits Identify barriers to participation, e.g., traveling long distances to attend visits Potential challenges of the inclusion/exclusion criteria and endpoints	A protocol developed in collaboration with patients can make adjustments for logistic challenges, medical needs, operational practicalities, and data collection methods (e.g., technology solutions)
Provide input to other patient facing materials For example: Identify communication barriers, e.g., co-create patient materials	Easy to understand studies can lead to faster enrollment, retention of participants, greater compliance with study requirements, building of trust, and addressing the needs of the intended participant population
Diversity of design For example: Provide insights on ways to gain more diverse participation in clinical studies, what challenges there may be to enrollment Inform a wider spectrum of Healthcare Professionals (HCPs) about the clinical activity, potentially expanding access to a diverse population Identify opportunities to reduce the burden of study design elements for participants	Develop proactive strategies to enable more diverse populations and perspectives to participate in clinical studies

## What is Next for Quality in Pharmaceutical Development?

ICH E8 furthers the concepts of quality by design, cementing it into a guidance adopted by many regulatory authorities and across the industry. Looking ahead, the resulting risk-based approaches to quality may benefit from new technologies, new data sources, and use of predictive analytics to proactively anticipate risks, further focusing resources and making a quality management system even more effective. With the additional areas of emphasis, it may also be time to refresh previously developed tools to reflect the broader bespoke perspectives introduced by ICH E8 and the new version of ICH E6.

## Appendix 1

### ICH Guidelines Connected by E8

As represented in Fig. 1.

**Table:**

ICH Guideline Reference	ICH Guideline Title
E6	Good Clinical Practice
Design Analysis	
M11	Clinical electronic Structured Harmonised Protocol (CeSHarP)
E4	Dose–Response Studies
E9	Statistical Principles for Clinical Trials
E10	Choice of Control Groups in Clinical Trials
E17	Multi-Regional Clinical Trials
E20	Adaptive Clinical Trials
Conduct & Reporting	
E3	Clinical Study Reports
Safety Reporting	
E1	Clinical Safety for Drugs used in Long-Term Treatments
E2A–E2F	Pharmacovigilance
E14	Clinical Evaluation of QT
E19	Safety Data Collection
Populations	
E5	Ethnic Factors
E7	Clinical Trials Geriatric Population
E11–E11A	Clinical Trials in Pediatric Population
E12	Clinical Evaluation by Therapeutic Category
Genetics/Genomics	
E15	Definitions in Pharmacogenetics/Pharmacogenomics
E16	Qualification of Genomic Biomarkers
E18	Genomic Sampling

## Footnotes

1 ICH E8 R1 General Considerations for Clinical Studies.

2 ICH E8 R1 General Considerations for Clinical Studies.

3 ICH E6 R2 Good Clinical Practice.

4 MHRA Blog: Regulators’ experience of clinical trials during the Covid-19 pandemic (Part 3)—looking forward (Gail Francis, Martin O'Kane, Kirsty Wydenbach and Lisa Campbell, Posted on:18 February 2022).

## Glossary

Term: Definition
Critical to Quality Factors (CtQs): Critical to Quality Factors (CtQs) are a set of elements that should be identified prior to the start of an activity, which can potentially have an impact on quality.
Critical Thinking: Critical Thinking is the proactive and ongoing analysis of available information, knowledge, and viewpoints to ensure an informed outcome.
Fit for Purpose: Fit for Purpose is whether the outcome addresses the research question while protecting participants and generating reliable results.
Open Dialogue: Open Dialogue is a proactive and ongoing approach to empower transparent and inclusive conversations with all stakeholders to achieve appropriate design and conduct.
Operational Feasibility: Operational Feasibility is a determination prior to the start of an activity and when there are changes that the activity can be conducted as designed.
Quality by Design (QbD): Quality by Design (QbD) sets out to ensure that quality is driven by proactively identifying and designing, with multiple stakeholders, the key aspects that require control to ensure protection of study participants, and reliability and/or interpretability of study results.
Stakeholder Engagement: Stakeholder Engagement is the proactive and ongoing process of ensuring that the interests of individuals who may be impacted or can influence quality are considered.

